# Taming the Rash: A Dermatological Case Report on Effective Treatments for Refractory Folliculitis

**DOI:** 10.7759/cureus.90835

**Published:** 2025-08-23

**Authors:** Rohit Sharma, Priyal Bharadwaj

**Affiliations:** 1 Microbiology, University of California Los Angeles, Los Angeles, USA; 2 Medicine, University of Kota, Kota, IND

**Keywords:** antibiotics, bacterial folliculitis, inflammation, pustules, steroids

## Abstract

Bacterial folliculitis, commonly associated with *Staphylococcus aureus*, often stems from trauma to hair follicles, such as during waxing, and can be exacerbated by improper aftercare that triggers inflammation and skin damage. This report describes a 23-year-old man with widespread folliculitis, mainly concentrated in the lower back, with involvement of the arms and entire back, post-waxing, aggravated by alcohol and soap use. He initially experienced high fever, trouble sleeping, discomfort when lying down, and strong itching, with the fever subsiding after a couple of days. He self-medicated with ibuprofen and diphenhydramine for five days without relief, followed by ineffective prescribed treatments with mupirocin and hydrocortisone. Adding oral azithromycin and Tend Skin® solution (isopropyl alcohol, water, propylene glycol, acetylsalicylic acid, cyclomethicone, glycerin, polysorbate 80) brought significant symptom relief, reduced pustule size, and less exudate within days. This case highlights the efficacy of combined systemic and topical therapies for refractory folliculitis and underscores the importance of proper post-waxing care to curb inflammation and skin damage.

## Introduction

Folliculitis, an inflammation of hair follicles, is a prevalent dermatological condition often triggered by bacterial infection, with *Staphylococcus aureus*, a common component of normal skin and nasal flora, as the primary suspected pathogen [1]. This condition can arise from various etiologies, including physical trauma, chemical irritation, or microbial colonization, with hair removal practices like waxing being a frequent precipitant due to disruption of the skin's protective barrier [2,3]. Folliculitis ranges from superficial to deep forms, presenting as erythematous papules, pustules, or nodules, often accompanied by pain, intense pruritus, and, in some cases, systemic symptoms like fever or sleep disturbances due to discomfort [1,4,5]. Its prevalence is notable in both community and clinical settings, with studies estimating that up to 20% of patients undergoing cosmetic procedures like waxing may develop post-procedure infections, particularly in hair-rich areas like the lower back, arms, and legs [6,7]. Untreated bacterial folliculitis can worsen, leading to increased lesion severity, abscess formation, or systemic complications such as cellulitis or bacteremia, especially in cases with delayed treatment or improper self-medication [4,8].

Waxing, a widely used hair removal method, involves mechanical epilation that causes micro-abrasions, enabling opportunistic pathogens like *S. aureus* to invade hair follicles [1,2]. Improper aftercare, such as alcohol-based products or harsh soaps, exacerbates this risk by causing epidermal irritation, dryness, and inflammation, which compromise the skin barrier and promote bacterial proliferation [3,6,9]. These factors can transform mild folliculitis into a refractory condition, resistant to standard topical therapies, and may intensify symptoms like itching or discomfort, particularly when lying on affected areas [4,5,10]. Beyond bacterial causes, folliculitis may result from fungal, viral, or non-infectious etiologies, but bacterial folliculitis due to *S. aureus* remains the most common form encountered in primary care [1,11]. The rising incidence of *methicillin-resistant S. aureus* (MRSA) further complicates management, necessitating careful antibiotic selection [12,13]. Patients often attempt self-treatment with over-the-counter agents like nonsteroidal anti-inflammatory drugs (NSAIDs) or antihistamines, which may temporarily alleviate symptoms like fever or itching but fail to address the underlying infection, potentially worsening the condition [4,8]. Topical antibiotics like mupirocin are standard for mild cases, but deeper or widespread infections may require systemic antibiotics and specialized topical agents [4,10]. This report describes the successful management of refractory bacterial folliculitis in a young man after waxing, using oral azithromycin and Tend Skin® solution (isopropyl alcohol, water, propylene glycol, acetylsalicylic acid, cyclomethicone, glycerin, polysorbate 80) after initial treatment failures. It highlights the importance of etiology-guided therapy, the risks of untreated bacterial infections, and the need for patient education to reduce inflammation, skin damage, and recurrence risk [6,14].

## Case presentation

A 23-year-old man visited his general practitioner with a one-week history of a painful, intensely itchy rash across his arms and entire back, mainly concentrated in the lower back. Written informed consent was obtained from the patient for the publication of this case report and the accompanying photograph. The rash emerged two days after a salon waxing session, during which he used isopropyl alcohol and soap for aftercare. He reported worsening redness, pustule formation, high fever, trouble sleeping, and discomfort when lying down, suggesting a significant infection. The fever subsided after a couple of days, but the itching and discomfort persisted. For five days before seeking medical care, he self-medicated with ibuprofen (400 mg three times daily) and diphenhydramine (25 mg at bedtime), alongside soap and water cleansing, without relief. The medical history was unremarkable, with no reported allergies or chronic conditions, with the exception of Sulfa drugs.

**Figure 1:**
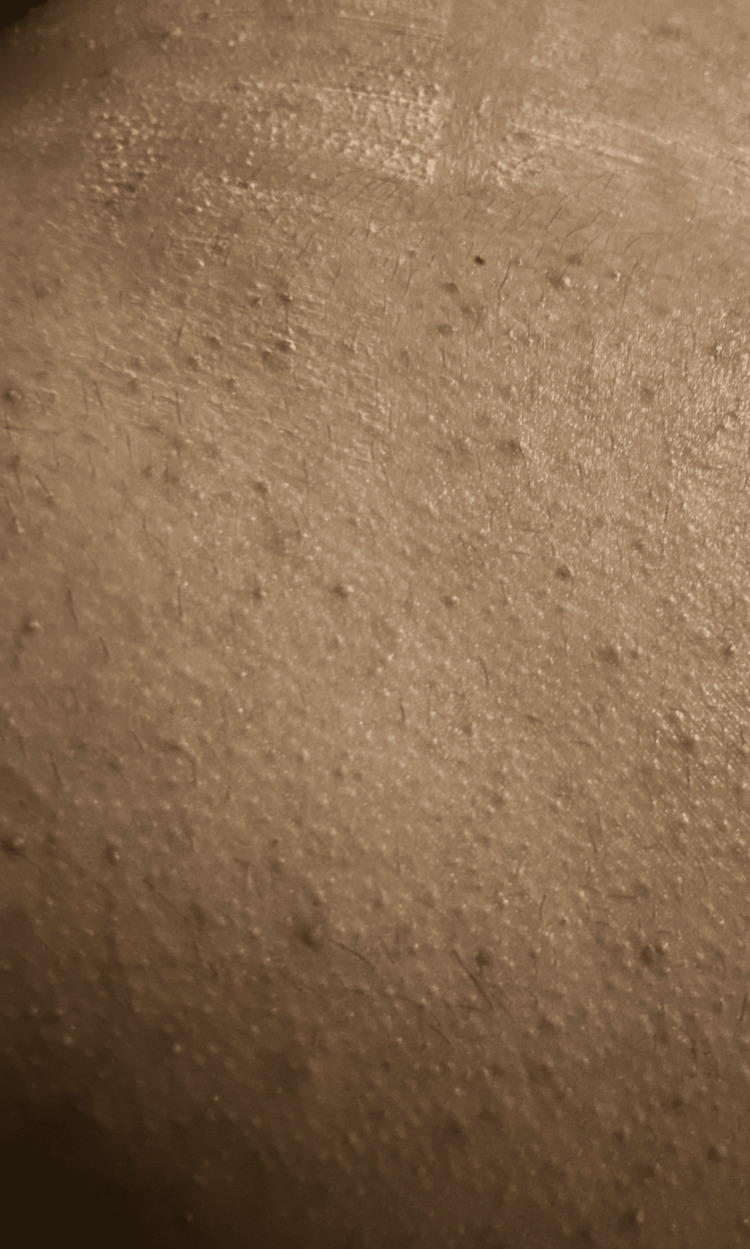
Erythematous papules and pustules on the lower back

Examination revealed multiple erythematous papules and pustules with purulent discharge, centered around hair follicles, spanning both arms and the entire back, with the most pronounced concentration in the lower back, covering roughly 20% of his body surface area. Scratches from irritation were also seen. No lymphadenopathy was observed (Figure 1).

The most likely diagnosis was bacterial folliculitis, given the clinical presentation and systemic symptoms, with *Staphylococcus aureus* suspected as the likely pathogen [1,5]. Other possible causes, such as Malassezia folliculitis or gram-negative folliculitis, were considered less likely given the clinical course. The presence of high fever and purulent pustules favored bacterial folliculitis over Malassezia folliculitis, which typically lacks systemic symptoms. A bacterial culture was not performed, as the etiology and clinical findings strongly supported a bacterial cause, though this represents a limitation of the diagnostic workup.

The patient was initially prescribed mupirocin 2% topical ointment and hydrocortisone 1% cream, both applied twice daily to address pain, itching, and inflammation. After five days, the pain, pustules, discharge, and itching persisted, with no notable improvement in the lower back's dense lesions. Treatment was escalated to include oral azithromycin (500 mg on day 1, followed by 250 mg daily for 4 days) and Tend Skin® solution (containing isopropyl alcohol, water, propylene glycol, acetylsalicylic acid, cyclomethicone, glycerin, polysorbate 80), applied twice daily with a cotton pad. He was instructed to stop alcohol-based aftercare and use a gentle, non-comedogenic cleanser to prevent further inflammation and skin damage [3,6].

**Figure 2:**
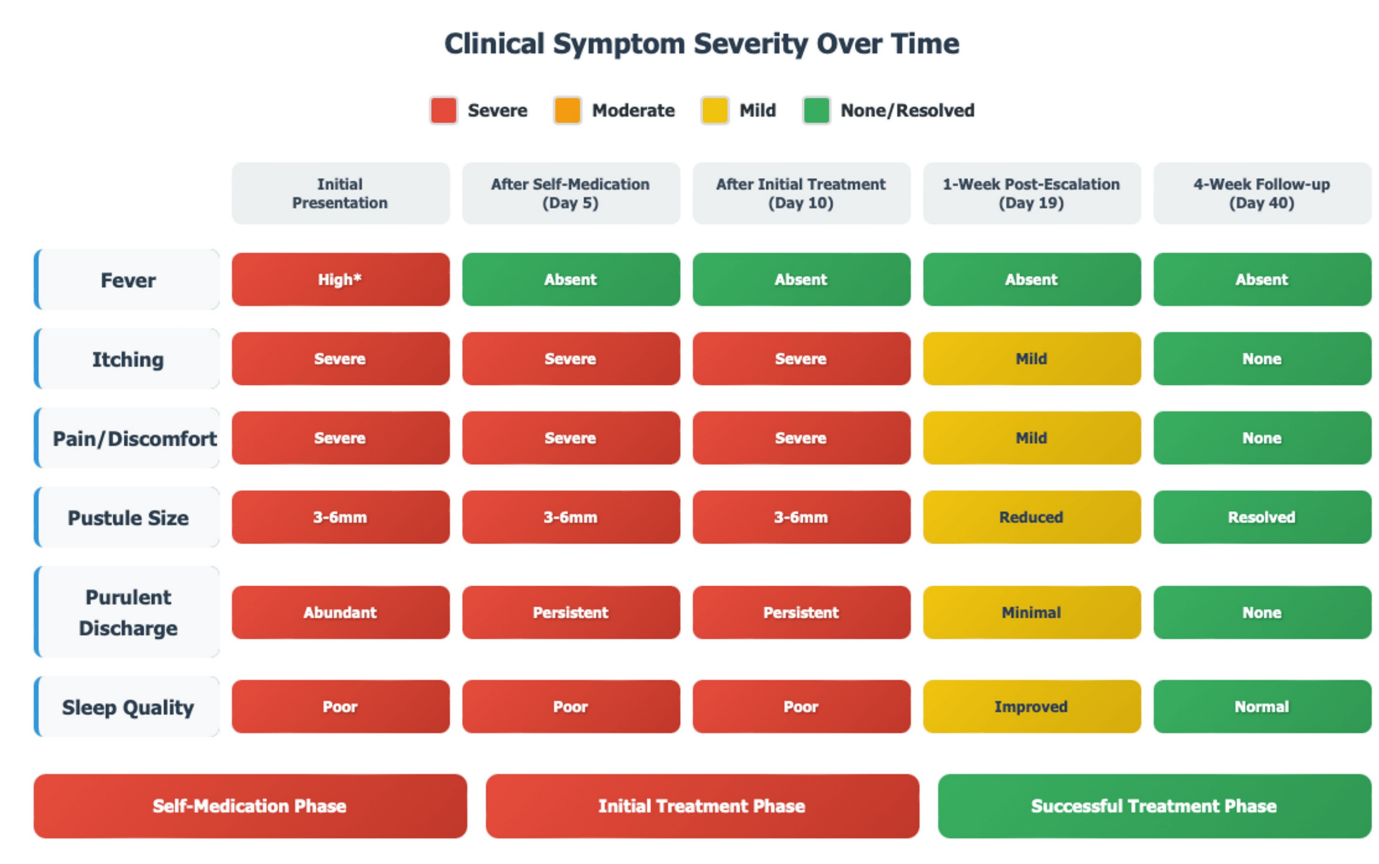
Symptom and treatment response Original work of the author

**Figure 3:**
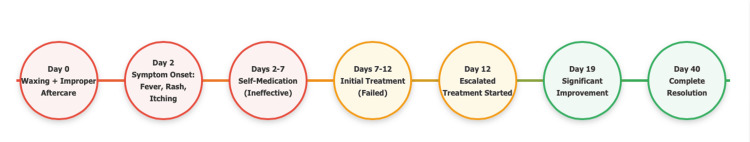
Clinical timeline Original work of the author

At a one-week follow-up, the patient reported significant relief from pain (decreased from 7/10 to 3/10), itching (from 8/10 to 3/10), and discomfort when lying down, particularly in the lower back. Examination showed reduced pustule size (from an average of 3-5 mm to 1-2 mm) and exudate, with most lesions beginning to crust and heal, including those in the densely affected lower back. No side effects from azithromycin or Tend Skin® were reported. The patient was counseled on post-waxing hygiene, including avoiding alcohol-based products and using emollients to protect the skin barrier and prevent further inflammation and damage [14]. At a four-week follow-up, the folliculitis had fully resolved, with only mild post-inflammatory hyperpigmentation, most noticeable in the lower back, and no recurrence. Symptomatology and a clinical timeline are shown below (Figures 2, 3).

## Discussion

This case of bacterial folliculitis, majorly concentrated in the lower back following waxing, aligns with studies highlighting the role of *Staphylococcus aureus*, a normal skin and nasal flora constituent, in post-hair removal infections [1,2]. Research indicates that mechanical trauma from waxing creates entry points for *S. aureus*, consistent with the patient's presentation of dense pustules in the lower back, a hair-rich area prone to follicular occlusion [1,15]. The patient's initial symptoms, high fever, trouble sleeping, discomfort when lying down, and strong itching, mirrored findings describing systemic symptoms like fever in severe folliculitis cases, often resolving spontaneously but leaving persistent local symptoms if untreated [5]. The fever's subsidence after a couple of days suggests partial immune control, but the persistent itching and discomfort underscore the risks of untreated bacterial infections, which can progress to abscesses, cellulitis, or systemic complications [8]. The patient's use of alcohol and soap for aftercare likely exacerbated inflammation and skin damage, as studies report that harsh post-waxing products increase infection risk by disrupting the epidermal barrier [3,6,9].

The differential diagnoses considered included Malassezia folliculitis and gram-negative folliculitis. Malassezia folliculitis, caused by *Malassezia furfur*, typically presents as monomorphic papules and pustules on the chest, back, and shoulders in young adults, particularly in warm, humid conditions. The acute onset following waxing trauma, presence of fever, and nature of the lesions made bacterial folliculitis more likely. Gram-negative folliculitis, often caused by *Pseudomonas* or *Proteus* species, typically occurs in patients with prolonged antibiotic use or immunocompromise and was considered less likely given the patient's history and clinical presentation.

The failure of self-medication with ibuprofen and diphenhydramine for five days aligns with findings that NSAIDs and antihistamines alleviate symptoms like fever and itching but do not address bacterial causes, potentially allowing worsening of the infection [4,8]. Similarly, the ineffectiveness of mupirocin and hydrocortisone in this case is consistent with research showing that topical therapies alone often fail in deeper or extensive folliculitis, particularly in areas like the lower back with dense follicular involvement [10,11]. The escalation to oral azithromycin, a macrolide with antimicrobial and anti-inflammatory properties, proved effective, supporting studies highlighting azithromycin's efficacy in *S. aureus* skin infections due to its dual action [16]. This contrasts with research noting challenges with antibiotic resistance in *S. aureus* infections, suggesting that azithromycin's success in this case may reflect a non-resistant strain, though culture confirmation was not pursued [12].

The use of Tend Skin® solution, containing acetylsalicylic acid, isopropyl alcohol, and glycerin, aligns with reports describing the efficacy of exfoliating and antimicrobial topicals in managing post-waxing folliculitis by clearing occluded follicles and reducing bacterial load [6]. Unlike the patient's initial alcohol-heavy aftercare, which likely worsened inflammation [9], Tend Skin's controlled application minimized irritation, supporting skin barrier repair. This case's success with Tend Skin® contrasts with studies focusing on moisturizing agents alone, suggesting that its multi-component formulation may offer unique benefits [3]. The lower back's prominence in this case reflects the heightened susceptibility of hair-dense areas to refractory infections, necessitating targeted therapies [15].

The decision to forgo bacterial culture aligns with studies supporting etiology-guided therapy in clear-cut cases of waxing-related folliculitis, though others advocate for cultures in refractory cases to rule out MRSA [1,13]. However, this represents a significant limitation of this case, as bacterial culture and antimicrobial sensitivity testing would have provided definitive pathogen identification and assessment of antibiotic resistance patterns, including differentiation between *methicillin-sensitive S. aureus* (MSSA) and methicillin-resistant *S. aureus* (MRSA). This limits the generalizability of the treatment approach, as the effectiveness of azithromycin could vary based on local resistance patterns. Additional limitations include the single-case design limiting broader applicability, the absence of standardized outcome measures for symptom assessment, and the inability to determine individual contributions of concurrent interventions (systemic antibiotic, topical treatment, and patient education) to the overall treatment success. The absence of recurrence in this case contrasts with reports of higher recurrence rates in patients without proper post-waxing care education [7]. The patient's education on avoiding alcohol-based products was critical in preventing further inflammation and recurrence [14]. Untreated or inadequately treated folliculitis can lead to worsening symptoms, including scarring or systemic spread, particularly in patients with initial systemic symptoms like fever [17]. This case's success with azithromycin warrants cautious use, as studies highlight the risk of resistance in regions with prevalent MRSA, suggesting alternatives like doxycycline in resistant cases [18]. The tolerability of Tend Skin®, with no reported adverse effects, supports its potential as a safe adjunctive therapy.

## Conclusions

This case illustrates the successful management of refractory bacterial folliculitis, majorly concentrated in the lower back, following waxing, using oral azithromycin and Tend Skin® solution (isopropyl alcohol, water, propylene glycol, acetylsalicylic acid, cyclomethicone, glycerin, polysorbate 80) after initial treatment failures, including five days of self-medication with ibuprofen and diphenhydramine. The patient's initial symptoms of high fever, trouble sleeping, discomfort when lying down, and strong itching, with the fever subsiding after a couple of days, highlight the significant inflammatory and infectious burden of untreated folliculitis. The persistence of symptoms despite self-medication and initial topical therapies underscores the limitations of symptom-focused treatments for bacterial infections. The rapid resolution achieved with azithromycin and Tend Skin® emphasizes the importance of combining systemic antibiotics with targeted topical treatments to address both infection and inflammation, particularly in areas with dense follicular involvement like the lower back. Patient education on proper post-waxing care, including avoiding alcohol-based products and using emollients to protect the skin barrier, was pivotal in preventing further inflammation and skin damage, ensuring complete recovery without recurrence.

The broader implications of this case highlight the need for clinicians to recognize the potential severity of folliculitis following cosmetic procedures like waxing, especially when compounded by improper aftercare or delayed treatment. Untreated bacterial infections can escalate, leading to worsening symptoms such as abscess formation, cellulitis, or systemic complications, particularly in patients presenting with systemic signs like fever. General practitioners and dermatologists should consider systemic antibiotics for refractory cases and prioritize patient counseling to prevent complications. The role of *S. aureus* as normal skin flora underscores the value of etiology-guided therapy when diagnostic resources are limited, though cultures remain essential for confirming pathogens in complex cases. Further research is needed to optimize treatment protocols, evaluate the efficacy of topical agents like Tend Skin® in larger cohorts, and develop standardized post-waxing care guidelines to reduce the incidence of folliculitis and related skin infections, ultimately improving patient outcomes in cosmetic dermatology.
